# FDG PET/CT and MR imaging of intramuscular myxoma in the gluteus maximus

**DOI:** 10.1186/1477-7819-10-132

**Published:** 2012-06-30

**Authors:** Jun Nishio, Masatoshi Naito

**Affiliations:** 1 Department of Orthopaedic Surgery, Faculty of Medicine, Fukuoka University, 7-45-1 Nanakuma, Jonan-ku, Fukuoka, 814-0180, Japan

**Keywords:** FDG, Intramuscular myxoma, MRI, PET/CT

## Abstract

Intramuscular myxoma is a rare benign soft tissue tumor which may be mistaken for other benign and low-grade malignant myxoid neoplasms. We present the case of a 63-year-old woman with an asymptomatic intramuscular myxoma discovered incidentally on a whole-body F-18 fluorodeoxyglucose (FDG) positron emission tomography (PET)/computed tomography. PET images showed a mild FDG uptake (maximum standardized uptake value, 1.78) in the left gluteus maximus. Subsequent magnetic resonance (MR) imaging revealed a well-defined ovoid mass with homogenous low signal intensity on T1-weighted sequences and markedly high signal intensity on T2-weighted sequences. Contrast-enhanced MR images showed heterogeneous enhancement throughout the mass. The diagnosis of intramuscular myxoma was confirmed on histopathology after surgical excision of the tumor. The patient had no local recurrence at one year follow-up. Our case suggests that intramuscular myxoma should be considered in the differential diagnosis of an oval-shaped intramuscular soft tissue mass with a mild FDG uptake.

## Background

Intramuscular myxoma is a rare benign soft tissue tumor of unknown origin. It usually occurs as an isolated lesion. The coexistence of intramuscular myxoma and skeletal fibrous dysplasia is known as Mazabraud’s syndrome [1]. To the best of our knowledge, F-18 fluorodeoxyglucose (FDG) positron emission tomography (PET)/computed tomography (CT) findings of intramuscular myxoma have been mentioned in one prior publication [2]. Here, we describe a case of intramuscular myxoma with a mild FDG uptake and discuss its clinicopathologic and radiologic features.

## Case presentation

**Figure 1:**
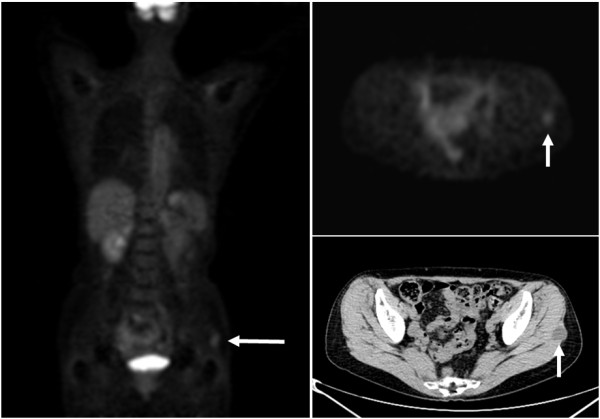
**FDG PET images demonstrate an increased uptake in the left buttock.** Transaxial CT shows a 3.5 cm hypodense mass, with corresponding tracer uptake (arrows).

**Figure 2:**
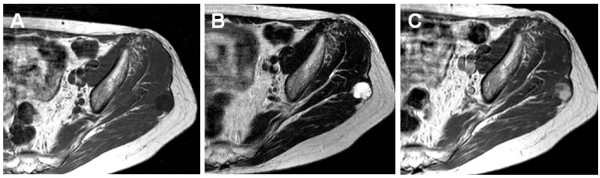
**Magnetic resonance imaging demonstrates a well-defined soft tissue mass in the left gluteus maximus.** The mass shows homogenous low signal intensity on T1-weighted images (**A**) and markedly high signal intensity on T2-weighted images (**B**). Axial T1-weighted images after gadolinium administration reveal heterogenous enhancement throughout the mass (**C**).

A 63-year-old previously healthy woman underwent whole body FDG PET/CT for cancer screening. FDG PET images demonstrated an increased uptake in the left buttock. The maximum standardized uptake value (SUV) was 1.78. CT showed a 3.5 cm hypodense mass, with corresponding tracer uptake (Figure 1). Physical examination did not show any abnormality in her left buttock. Laboratory findings were within normal limits. Subsequent magnetic resonance imaging (MRI) demonstrated a well-defined soft tissue mass in the left gluteus maximus. The mass showed homogenous low signal intensity on T1-weighted images (Figure 2A) and markedly high signal intensity on T2-weighted images (Figure 2B). A thin rim of higher signal intensity approaching that of fat was seen around the mass on T1-weighted images. Contrast-enhanced T1-weighted images revealed heterogenous enhancement throughout the mass (Figure 2C). Based on these findings, benign and low-grade malignant myxoid neoplasms were suspected, including intramuscular myxoma and myxoid liposarcoma.

**Figure 3:**
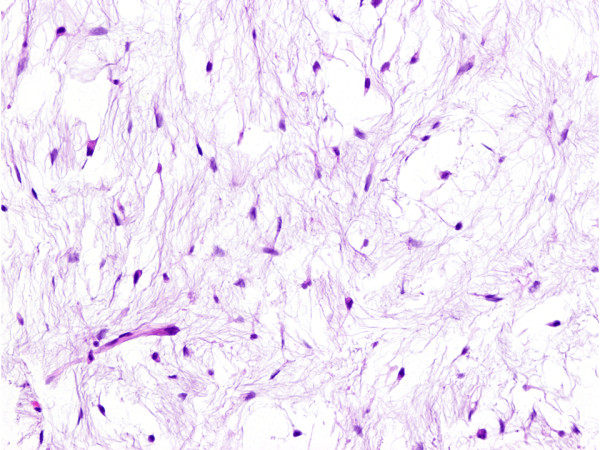
**Histologic finding of intramuscular myxoma.** The tumor is composed of bland spindle and stellate shaped cells in an abundant myxoid stroma.

An open biopsy was performed. The tumor was composed of bland spindle and stellate shaped cells that were widely separated by myxoid stroma (Figure 3). No mitotic figures were seen. The tumor cells were immunohistochemically positive for vimentin and CD34, but negative for S-100 protein. These findings were consistent with intramuscular myxoma. We then performed a marginal excision of the tumor. Grossly, the excised tumor showed a gelatinous cut surface with a small cyst-like space, measuring 3.5 × 2.0 × 1.6 cm. All tissues were examined histologically by using sections stained with hematoxylin and eosin. No hypercellularity, hypervascularity, or mitotic figures were identified. The MIB-1 labeling index was less than 1%. These findings confirmed the diagnosis of intramuscular myxoma. The postoperative course was uneventful, and the patient is doing well without local recurrence one year after the surgery.

## Discussion

Intramuscular myxoma has a peak incidence in the fourth to sixth decades of life with a female predominance. It typically presents as a slowly growing, painless mass in the large muscles of the thigh, shoulder, buttock, and upper arm [3,4]. Marginal excision is the treatment of choice [5]. Local recurrence is rare, and there is no risk for metastasis. Recently, *GNAS1* mutations have been identified in intramuscular myxoma with and without fibrous dysplasia [6,7]. Interestingly, Willems *et al*. [8] reported that *GNAS1* mutation analysis can be helpful to distinguish intramuscular myxoma from low-grade myxofibrosarcoma in selected cases.

The gross appearance is characteristic and the tumor has a mucoid, gelatinous cut surface with thin fibrous septa. Histologically, intramuscular myxoma is composed of bland spindle and stellate shaped cells in an abundant myxoid stroma. Fluid-filled cystic spaces are seen occasionally. Mitotic activity and cellular pleomorphism are usually absent or minimal. Some intramuscular myxomas show focal areas of hypercellularity and hypervascularity, which may be confused with several myxoid soft tissue sarcomas such as myxoid liposarcoma, low-grade myxofibrosarcoma, and low-grade fibromyxoid sarcoma [9]. Immunohistochemically, the cells stain positively for vimentin and show variable staining for CD34 and actin. Immunostain for S-100 protein is typically negative.

Intramuscular myxoma is shown as a homogeneous low-attenuating mass on CT [4]. MRI reveals a well-defined ovoid mass exhibiting low signal intensity relative to skeletal muscle on T1-weighted images and markedly high signal intensity on T2-weighted images. Contrast-enhanced CT and MRI studies demonstrate heterogeneous internal enhancement and/or peripheral enhancement with occasional fine internal septa. MRI may show surrounding fat rim or cap and surrounding muscle edema [4,10-12], as in our case. These features are strongly suggestive of intramuscular myxoma, but it might be difficult to distinguish cellular/intramuscular myxoma from myxoid liposarcoma in some cases.

FDG PET is increasingly used for the detection and management of soft tissue tumor. In the present case, PET revealed an increased uptake of FDG with a maximum SUV of 1.78. Only one case of a positive FDG PET in intramuscular myxoma has been reported so far [2]. In that report, the maximum SUV was 1.8 and FDG activity was predominantly located in the periphery of the lesion. Moreover, an appearance of Mazabraud’s syndrome on FDG PET has been described and the SUV range for the intramuscular myxoma lesions was between 1.3 and a maximum of 2.6 [13]. Our case and others suggest that intramuscular myxoma has a mild FDG uptake. The mechanism of uptake is uncertain but may reflect a varying proportion of metabolically active cells. On the other hand, a previous FDG PET study demonstrated that the mean SUV for myxoid liposarcoma was 2.15 [14]. Based on these findings, FDG PET appears to be insufficient as a screening method for differential diagnosis between intramuscular myxoma and pure myxoid liposarcoma.

## Conclusions

We report the second case describing FDG PET/CT imaging of intramuscular myxoma. This tumor should be considered in the differential diagnosis of an oval-shaped intramuscular soft tissue mass with a mild FDG uptake.

## Consent

Written informed consent was obtained from the patient for publication of this case report and any accompanying images. A copy of the written consent is available for review by the Editor-in Chief of this journal.

## Abbreviations

CT, Computed tomography; FDG, Fluorodeoxyglucose; MRI, Magnetic resonance imaging; PET, Positron emission tomography; SUV, Standardized uptake value.

## Competing interests

The authors declare that they have no competing interests.

## Authors’ contributions

JN managed the patient and drafted the manuscript. MN helped to draft the manuscript. Both authors read and approved the final manuscript.
